# Insulin dysregulation in a population of Finnhorses and associated phenotypic markers of obesity

**DOI:** 10.1111/jvim.15782

**Published:** 2020-06-17

**Authors:** Justin R. Box, Cathy M. McGowan, Marja R. Raekallio, Anna K. Mykkänen, Harry Carslake, Ninja P. Karikoski

**Affiliations:** ^1^ Department of Equine and Small Animal Sciences, Faculty of Veterinary Medicine University of Helsinki Helsinki Finland; ^2^ Institute of Veterinary Science University of Liverpool Neston United Kingdom

**Keywords:** EMS, equine, laminitis, OST

## Abstract

**Background:**

Obesity and insulin dysregulation (ID) predispose horses to laminitis. Determination of management practices or phenotypic markers associated with ID may benefit animal welfare.

**Objectives:**

Determine ID status of a population of Finnhorses using an oral sugar test (OST) and compare phenotypes and management factors between ID and non‐ID Finnhorses.

**Animals:**

One hundred twenty‐eight purebred Finnhorses ≥3 years of age.

**Methods:**

Owners were recruited using an online questionnaire regarding signalment, history, feeding, and exercise of their horses. Selected contributing stables within a predefined area were visited. Phenotypic markers of obesity and the weight of each horse were recorded. After fasting overnight, horses received 0.45 mL/kg corn syrup PO. Serum samples before and at 60 and 90 minutes after syrup administration were analyzed for insulin by chemiluminescent assay. Horses met ID criteria if insulin concentrations were ≥33 μIU/mL at T0, ≥66 μIU/mL at T60 or T90 or some combination thereof. Associations between phenotypic markers, feeding and exercise variables, and ID were examined using mixed effects logistic regression modeling.

**Results:**

Several phenotypic markers of obesity were significant on univariable analysis but in the final multivariable model, only obesity (body condition score  ≥8) was associated with ID (*P* = .04). Over half of the horses (60% [95% confidence interval (CI), 51%‐68%]) were considered overweight or obese whereas 16% (95% CI, 10%‐23%) were classified as having ID.

**Conclusions and Clinical Importance:**

Because obesity is associated with ID in cold‐blooded type horses, objective monitoring of phenotypic markers by owners may be beneficial for health outcomes.

**Abbreviations**
AUC: area under the curve
BCS: body condition score
BW: body weight
CI: confidence interval
CNS: cresty neck score
EMS: equine metabolic syndrome
ID: insulin dysregulation
IS: insulin sensitivity; OR, odds ratio
OR: odds ratio
OST: oral sugar test

## 1 INTRODUCTION

Obesity is a major risk factor for insulin dysregulation (ID) and a substantial health problem among equine populations worldwide.^1^, ^2^, ^3^ Insulin dysregulation is defined as any combination of basal hyperinsulinemia, postprandial hyperinsulinemia (in response to dynamic testing), or insulin resistance,^4^ and is an important predisposing factor for laminitis, a painful hoof condition in horses that can lead to loss of use, chronic lameness, and even death.^5^ Insulin dysregulation and generalized or regional adiposity are features of equine metabolic syndrome (EMS) and can be used as predictors of laminitis.^6^, ^7^ Although not all obese animals have ID (and vice versa), dynamic endocrine testing and appropriate weight management of overweight animals are recommended to decrease the possible risk for laminitis.^6^, ^8^

Dynamic testing is the preferred method of determining ID in horses and is more sensitive than basal testing alone.^6^ The oral sugar test (OST) has been described in several studies^9^, ^10^, ^11^ as an ideal, replicable method for dynamic ID testing at different doses (0.15‐0.45 mL/kg). It has been shown to be repeatable using binary outcomes^10^ and is comparable to more invasive tests^9^; therefore, it is a practical approach for on‐farm testing.

Prevalence of ID in horses has been shown to vary from 18% to 27% depending on the specified population.^2^, ^12^, ^13^ Breed differences in ID have been identified. For example, ponies and Andalusian horses had significantly lower insulin sensitivity (IS) than did Standardbred horses.^14^ Additionally, many of the published cases of EMS have occurred in native British breeds. One study found that cases of primary endocrinopathic laminitis (induced by ID with or without pituitary pars intermedia dysfunction) were more likely to occur in native British ponies compared to native Nordic ponies, cold‐, warm‐, and hot‐blooded horses.^15^ However, in another study, cold‐blooded type ponies had increased risk of laminitis compared to warm‐blooded type ponies.^16^ The ID status of certain cold‐blooded equine populations, such as Finnhorses, has not been investigated.

The Finnhorse is a cold‐blooded horse originating from Northern European domestic horses. They are the only equine breed native to Finland and have been bred as a pure breed since 1907, when the studbook was founded. The registry recognizes 4 types of Finnhorse: racing trotter, riding and pleasure, working, and pony‐sized (www.hippos.fi).

Factors associated with increased obesity risk include management and exercise, primarily resulting from decreased physical activity and excess energy intake, although genetic and epigenetic factors also may play a role.^17^, ^18^ In humans, physical activity has been shown to improve IS, even in the absence of apparent weight loss.^19^ In studies of horses, exercise has been shown to decrease serum concentrations of inflammatory markers (serum amyloid A and haptoglobin)^20^ and improve ID, particularly when exercise was of moderate intensity.^21^, ^22^

In our study, we evaluated the ID status of a population of Finnhorses in southern Finland by determining insulin response to corn syrup OST. Our aim was to compare phenotypic markers of obesity and management factors between ID and non‐ID Finnhorses.

## 2 MATERIALS AND METHODS

### 2.1 Animals

The study protocol was approved by the National Animal Experimentation Board of Finland (ESAVI/6728/04.10.07/2017). Horses met study inclusion criteria if they were located within 150 km of Helsinki, were ≥3 years old, and had no clinical evidence or history of systemic inflammatory disease. A physical examination was performed on all horses by a veterinarian and any animals with fever (≥38.5°C), tachycardia, tachypnea, signs of systemic inflammatory disease, or any other potentially painful condition were excluded. An initial serum biochemistry profile and a CBC were performed on each horse to evaluate health status. Biochemical results were determined using a commercial biochemistry analyzer (Konelab 30 Clinical Chemistry Analyzer, ThermoFisher Scientific, Vantaa, Finland). The CBC was performed using an ADVIA 212io hematology analyzer (Siemens, Tarrytown, New York), and plasma fibrinogen concentration was determined using a heat precipitation method.^23^ Any animal with abnormal biochemical or CBC findings was excluded. Animals with previously diagnosed pituitary pars intermedia dysfunction also were excluded.

### 2.2 Questionnaire

A link to a web‐based questionnaire was advertised from September to December in 2017 in the University of Helsinki Faculty of Veterinary Medicine webpages seeking study enrollment by owners of purebred Finnhorses ≥3 years of age living within approximately 150 km of Helsinki. The Veterinary Medicine webpages provided owner resources and information about the hospital that were regularly accessed by equine owners. The questionnaire requested information about the signalment, history, feeding, exercise and previous and current diseases of each horse. Additionally, owners were asked to estimate their horse's body condition score (BCS, Henneke 1‐9 scoring system)^24^ and cresty neck score (CNS, Carter 0‐5 scoring system)^25^ with the help of illustrative figures. With regard to exercise, owners were asked to report their horse's main use (racing, draft, riding competition, pleasure riding, pet, breeding), estimate how many days per week on average they exercised their horse, how many days per week the horse was sweating during exercise, and how many hours per week the horse was exercised at walk, trot, and canter. Horses were grouped into either intense use (racing, draft, competition) or nonintense use (pleasure riding, pet, breeding). The hours per week spent trotting and cantering (trot + canter) were added together as a single analysis value. Cumulative exercise was calculated by adding walk, trot, and canter hours per week. Finally, the owners were asked to report the amount of roughage (kg) and concentrate (kg) their horse received each day. Concentrate was defined as any feed (commercially prepared or otherwise) given to the horse that was not a vitamin or mineral supplement or both or type of roughage. If owners reported a range, the upper limit value was used for analysis.

### 2.3 Sample size

A convenience sample of recruited horses that were ≥3 years old were selected for a stable visit based on their geographical location (within 150 km of the institution). Before the start of testing, sample size was calculated using the online Epitools sample size calculator^26^. Given the population size of approximately 20 000 Finnhorses, an expected incidence of 15% to 20%, a confidence interval (CI) of 95%, and a power of 80%, the calculated sample size was 150. Operations housing ≥5 Finnhorses initially were selected but premises with <5 horses later were included. All available horses at each stable that met inclusion criteria had OST performed.

### 2.4 Physical measurements

One of 2 trained veterinarians (Justin R Box, Ninja P Karikoski) performed the physical measurements, including phenotypic markers of obesity and hoof wall changes. Phenotypic markers of obesity included BCS, CNS, and supraorbital fat pads. Assessment of macroscopic hoof wall changes that were indicative of laminitis included divergent growth rings, white line separation, and dropped soles.

The following physical measurements were obtained using a weight tape designed for horses (Virbac Animal Health): weight, heart‐girth, widest part of the abdomen, and neck circumference (midpoint of the neck). Additionally, BCS and CNS were assessed.^24^, ^25^ Horses with BCS of 7 were considered over‐conditioned^3^ and classified as obese if their BCS was ≥8. Horses were considered to have a cresty neck if CNS was ≥3. Supraorbital fat pads were graded on a scale of 0‐3; 0 being deep/concave and 3 being rounded/convex.

### 2.5 Basal blood samples

Basal blood glucose concentrations were determined using lithium‐heparin blood (Vacuette LH, Greiner Bio‐One, Kremsmünster, Austria) immediately after sampling using a handheld veterinary glucometer (AlphaTRAK II, Zoetis, North Chicago, Illinois).

Blood for adrenocorticotropic hormone (ACTH) concentration measurement was collected in 6‐mL EDTA tubes (Vacuette K2EDTA, Greiner Bio‐One, Kremsmünster, Austria) and kept cool until centrifugation (within 8 hours). The separated plasma was frozen and stored at −80°C until shipment on dry ice to the diagnostic laboratory. Analyses were performed in duplicate using a chemiluminescent immunoassay (Immulite 2000 XPi, The Philip Leverhulme Equine Hospital, Liverpool, UK). All animals with seasonally increased basal plasma ACTH concentrations were excluded from the study. The seasonally adjusted ACTH cutoff concentrations used for the study were 89.4 pg/mL for horses sampled in October and 35.2 pg/mL for horses sampled in November and December (Adams A., Abstract, International Equine Endocrinology Summit, 2017).

### 2.6 Oral sugar test

Oral sugar tests were performed either at the stables (n = 139) or in the University of Helsinki (n = 5) during a period from the last week of October 2017 through the second week of December 2017. The evening before the test, the horses were stalled and allowed to have a slice of dry hay or haylage (1‐2 kg) no later than 22:00. No grain or additional hay was allowed until after the OST was completed. All horses had access to water throughout the entire experiment. The OSTs were performed in the morning between 06:00 and 10:00. Horses were given 0.45 mL/kg body weight (BW) corn syrup PO (Karo Light, ACH Food Companies Inc, Cordova, TN) via 100‐ml dosing syringes. Karo Light contains, on average, 158 mg/mL of maltose and 198 mg/mL glucose, so that horses received a combined maltose and glucose dose of 160.3 mg/kg BW.^11^ For insulin concentration measurement, blood was collected into 6‐ mL serum tubes (Vacuette, Z serum clot activator, Greiner Bio‐One, Kremsmünster, Austria) before syrup administration and at 60 (T60) and 90 (T90) minutes thereafter. Blood was allowed to clot at ambient temperature for at least 60 minutes. Subsequently, all samples were centrifuged, serum separated within 8 hours, and stored at −80°C until shipment on dry ice to the laboratory for analysis. All samples were measured in duplicate using a chemiluminescent immunoassay (Immulite 2000 XPi, The Philip Leverhulme Equine Hospital, Liverpool, United Kingdom). The reportable range for the Immulite 2000XPi was 2‐300 μIU/mL. Our preliminary correlation studies have shown excellent correlation (*r* = .996) between the Immulite 2000XPi and the more commonly used Immulite 2000 (Carslake, H., unpublished data), but the 2000XPi reports consistently higher values than the 2000. Therefore, a higher cutoff concentration of 66 μIU/mL was used for our study instead of the suggested 40 μIU/mL. Horses were categorized as having ID based on insulin concentrations ≥33 μIU/mL at T0 or ≥66 μIU/mL at either T60 or T90 or both.

### 2.7 Statistical analysis

The area under the curve (AUC) was calculated for the insulin response (T0‐T90) using the trapezoidal method. The normality of each variable distribution was tested using the Shapiro‐Wilk test. Correlations among BCS, glucose, and insulin were tested using Spearman rank correlation with Bonferroni correction. Comparison of owner versus investigator BCS and CNS was performed using related samples Friedman's 2‐way analysis of variance by ranks.

To assess the effect of different covariates on ID, mixed effects logistic regression models, modeling the odds for occurrence of ID, were fitted. First, each covariate was separately modeled with the response, the model including ID status as a response, covariate as a fixed effect and cluster (stable) as a random effect (univariable analysis). Variables with *P* value <.2 were taken forward to multivariable analyses. Similar mixed effects logistic regression models as for the individual analyses were fitted. In all models, odds ratios (OR) for comparisons between groups for categorical covariates or increase of 1 unit in continuous or ordinal covariates with 95% CI and *P* values were estimated using contrasts from the same model. *P* values <.05 were considered statistically significant. Statistical analyses were performed at 4Pharma Ltd using SAS System for Windows, version 9.4 (SAS Institute Inc., Cary, North Carolina).

## 3 RESULTS

Two hundred thirty‐three owners completed the online questionnaire, representing 291 horses. One hundred forty‐four horses from 30 premises were selected for detailed examination and testing. Of the 144 horses sampled, 1 horse was excluded because of increased body temperature and 15 were excluded because of seasonally increased basal plasma ACTH concentrations. The remaining 128 horses consisted of 63 geldings (49%), 58 mares (45%), and 7 stallions (5%). Owners categorized the use of their horses in the following manner: 106 as “pleasure riding” horses, 7 as “riding competition” horses, 2 as “breeding” horses, 10 as “racehorses,” 2 as a “pet,” and 1 as a “draft horse.” Therefore, 18 horses were considered as experiencing intense use and 110 nonintense use.

The phenotypic marker results of the 128 horses are presented in Table 1. There were 77/128 (60%; 95% CI, 51%‐68%) overconditioned or obese horses, of which 35/128 (27%; 95% CI, 20%‐36%) were overconditioned and 42/128 (33%; 95% CI, 25%‐41%) were obese. The BCS given by the owners were significantly lower than the BCS given by the investigator (*P* < .001). The CNS given by the owners, however, were significantly higher than those given by the investigator (*P* < .001). Forty‐two horses (33%; 95% CI, 25%‐41%) were considered to have a cresty neck. Seventy‐three (57%; 95% CI, 48%‐65%) horses had visible growth rings on ≥1 hooves, but none of the horses had divergent hoof rings, white line separation, or dropped soles indicative of a history of laminitis. Six horses had a history of laminitis diagnosed by a veterinarian and 2 additional horses had historical owner‐suspected but not veterinarian‐confirmed laminitis.

**TABLE 1:** Descriptive data of phenotypic markers, endocrine values and management factors reported by the equine owner, investigator or both in insulin dysregulated (ID, n = 20) and non‐ID (n = 108) Finnhorses

Variable	Non‐ID (n = 108)	ID (n = 20)
Age (y)	9.0 (5‐13)	12.0 (7.25‐16)
BCS investigator (1–9)	7.0 (6‐7.5)	8.0 (6.25‐8)
BCS owner (1–9)	6.0 (5‐7)	7.0 (6‐7)
Body weight (kg)	553 (504‐601)	585 (541‐626)
CNS researcher (0‐5)	2.0 (1‐3)	2.5 (2‐3)
CNS owner (0‐5)	2.0 (2‐3)	3.0 (2‐3)
Supraorbital fat (0‐3)	1.0 (0‐1)	1.0 (1‐1.5)
Heart‐girth (cm)	190 (184‐196)	195 (190‐199)
Widest part of abdomen (cm)	214 (205‐222)	218 (213‐225)
Neck circumference (cm)	110 (104‐114)	110 (104‐115)
Triglycerides (mmol/L)	0.23 (0.19‐0.27)	0.25 (0.17‐0.34)
Basal glucose concentration (mmol/L)	6.0 (5.5‐6.4)	6.4 (6.1‐6.7)
Amount of roughage (g/kg BW)	19.7 (15.7‐23.9)	18.2 (16.8‐21.6)
Amount of concentrates/grain (g/kg BW)	8.43 (.0‐18.1)	7.35 (1.08‐19.1)
Exercise (d/wk)	5.0 (3.0‐5.0)	5.0 (3.0‐5.0)
Sweating during exercise (d/wk)	3.0 (1.0‐3.0)	1.0 (1.0‐3.0)
Trot + canter (h/wk)	3.0 (1.0‐4.0)	2.0 (.25‐2.75)
Cumulative exercise (h/wk)	5.0 (3.0‐6.0)	3.5 (2.25‐4.75)

*Note:* Values are reported as median and (interquartile range).

Abbreviations: BCS, body condition score; CNS, cresty neck score.

### 3.1 Oral sugar test

No adverse events were noticed by the investigators during the OST or reported by the owners after the study. All but 1 of the horses readily accepted the PO dosing of the syrup. The 1 horse that refused the dosing syringe consumed all of the syrup (within 1‐2 minutes) after the entire volume was ejected into the horse's empty food bucket. In total, 20/128 (16%; 95% CI, 10%‐23%) horses met the criteria for ID (Table 2). Of these, only 1 animal had increased insulin concentration at T0, 9 at T60, and 19 at T90. Eight horses had increased insulin concentrations at both T60 and T90. Seven of the 8 horses with owner‐reported history of laminitis were categorized in the ID group.

**TABLE 2:** Group‐wise comparison of insulin values T0, T60, T90, and area under the curve (AUC) divided into insulin dysregulated (ID) and non‐ID in response to an oral sugar test

Variable	All (n = 128)	Non‐ID (n = 108)	ID (n = 20)	Range all (n = 128)
Insulin T0 (μIU/mL)	<2.0 (<2.0)	<2.0 (<2.0)	<2.0 (<2.0‐6.6)	<2.0‐35.4
Insulin T60 (μIU/mL)	11.4 (5.6‐28.8)	9.4 (5.2‐17.6)	59.1 (43.2‐79.6)	<2.0‐254.5
Insulin T90 (μIU/mL)	14.0 (7.9‐40.8)	11.4 (6.9‐20.8)	87.0 (73.1‐114.6)	<2.0‐446.5
Insulin AUC	771 (381‐1847)	609 (368‐1097)	4017 (3324‐5002)	0‐19 211

*Note:* Values reported are the median and interquartile range.

Body condition scores correlated significantly with insulin T0 (*ρ* = .253, *P* = .04), T60 (*ρ* = .309, *P* < .01), T90 (*ρ* = .270, *P* = .02), and AUC (*ρ* = .305, *P* < .01). Basal glucose concentration correlated significantly with insulin T60 (*ρ* = .267, *P* = .03) and AUC (*ρ* = .261, *P* = .03). No other significant correlations were detected among BCS, glucose, and insulin.

Of the 42 obese horses, only 11 (26%; 95% CI, 15%‐41%) met ID criteria, and of the 35 overconditioned horses, only 4 (11%; 95% CI, 5%‐26%) met ID criteria. Five (4%; 95% CI, 2%‐9%) non‐overconditioned or obese horses were categorized as having ID.

Several variables were found to be significant in univariable analysis (Table 3) and these were moved forward into a multivariable model. However, because several significant obesity‐related variables were found in univariable analysis that correlated with each other, the variables that were not affected by the horse's height (BCS, CNS, and obesity versus heart‐girth, weight, widest part of abdomen) were selected for multivariable analysis. Each of the obesity variables was analyzed separately in a multivariate model with the other 4 (age, sex, glucose, combined trot and canter hours/week) variables (3 separate models). Finally, only obesity (BCS ≥ 8) was shown to be associated with ID in multivariable models (OR, 3.29; 95% CI, 1.04%‐10.37%; *P* = .04).

**TABLE 3:** Results of univariable mixed effects logistic regression analysis of signalment, morphometric and metabolic variables in 128 Finnhorses accounting for clustering at stable level

Variable	Odds ratio	95% CI	*P* value
Age (y)	1.07	0.98‐1.17	.15
Sex (female versus male)	2.13	0.74‐6.10	.16
Usage (nonintense versus intense)	3.77	0.42‐33.55	.23
**BCS 1–9 (1 unit)**	**2.11**	**1.18‐3.77**	**.01**
**Obesity (yes/no)**	**4.24**	**1.45‐12.37**	**.009**
**CNS 0–5 (1 unit)**	**1.96**	**1.07‐3.59**	**.03**
Cresty neck (yes/no)	2.95	0.99‐8.33	.05
Supraorbital fat (1 unit)	1.51	0.73‐3.13	.27
**Weight (kg)**	**1.01**	**1.00‐1.02**	**.04**
Widest part of abdomen (cm)	1.05	1.00‐1.10	.05
**Heart‐girth (cm)**	**1.08**	**1.01‐1.16**	**.03**
Neck circumference (cm)	1.03	0.97‐1.09	.41
Basal glucose (mmol/L)	2.00	0.94‐4.25	.07
Triglycerides (mmol/L)	1.47	0.74‐2.95	.27
Amount of concentrates/grain (g/kg BW)	0.07	0.00‐8.35	.27
Amount of roughage (g/kg BW)	0.48	0.15‐1.55	.22
Exercise (d/wk)	1.08	0.77‐1.51	.67
Sweating during exercise (d/wk)	0.83	0.56‐1.23	.36
Trot + canter (h/wk)	0.75	0.53‐1.04	.08
Cumulative exercise (h/wk)	0.90	0.73‐1.11	.31

*Note:* Significant results are shown in bold.

Abbreviations: BCS, body condition score; CNS, cresty neck score.

## 4 DISCUSSION

In this population of Finnhorses in southern Finland tested between October and December in 2017, obesity was the only variable associated with ID in multivariable analysis. The risk for ID was 3.29 times higher in horses with BCS ≥8 than in horses with lower BCS. In addition, several phenotypic markers related to obesity (BCS, CNS, BW, heart‐girth, widest part of the abdomen) were found to be significant on univariable analysis. However, variables associated with feeding or exercise were not significant risk factors in this Finnhorse population. Small sample size could be a reason for the lack of association with these variables, with the majority of horses being overconditioned or obese and not undergoing intense exercise.

Not all obese or overconditioned horses had ID. In fact, most of the obese or overconditioned horses (81%) did not have ID. Supraorbital fat pad and neck circumference were not significant risk factors in univariable analysis. In 2 previous studies, ponies with ID (basal hyperinsulinemia) did not have significantly higher BCS or CNS than did ponies with normal insulin concentrations, and therefore the authors suggested that assessment of physical obesity parameters might not be an accurate predictor for ID in native pony breeds.^8^, ^12^ However, the majority of ponies in these studies were overconditioned or obese, which may have affected the results. In another more heterogenous group of ponies, CNS was positively associated with postprandial insulin concentration (oral glucose test), and ponies with a cresty neck had 5 times higher risk of having ID than did ponies with a normal neck.^7^ In addition, in a mixed equine population in the US, overconditioned and obese horses had significantly higher basal plasma insulin concentrations (indicative of ID) compared to optimally conditioned horses.^13^ Additionally, that study found breed differences in IS and insulin concentrations. Therefore, the association between ID and obesity indeed may be breed‐related, and this possibility should be taken into consideration when evaluating the status of and risks for ID in an individual horse.

Exercise was not shown to be a protective factor for ID in our study. However, none of the animals registered as trotting racehorses (n = 10) met the criteria of ID or had a history of laminitis, nor were any of them obese. Previously published studies indicate that moderate‐intensity, short‐term (45 min/d for 7 days)^21^ and long‐term (60 min/d for 1 month)^27^ training have been shown to improve ID in horses. Additionally, a recent study showed that ID was significantly improved by diet modification and low‐intensity exercise when compared with diet modification alone.^28^ The study also found that low‐intensity exercise without diet change was insufficient to improve IS despite decreases in total body fat mass. Another study demonstrated that even long‐term low‐intensity exercise, such as walking 2 hours twice daily for 3 months, did not improve IS although the animals lost weight during the research period.^29^ Therefore, light exercise alone, even if done regularly several hours per week, may not be sufficient to protect horses from ID. Accuracy in owner reporting may have been a factor in the nonsignificance of exercise data in our study. The questionnaire was designed to be as straightforward as possible, but some owners' perceptions regarding health, nutrition and exercise intensity may not have been realistic, as has been shown in some previous studies.^30^, ^31^

Owners underestimated their horses' BCS by 1 grade compared to the investigators, in agreement with previous study reports,^2^, ^32^, ^33^ which is relevant when assessing weight management of horses. Because BW, heart‐girth and widest part of the abdomen were taken with a standard, commercially available weight and measuring tape designed for horses, owners can record and track measurements of their horses without the need of a veterinarian or expensive equipment. Use of this tool allows owners of horses with ID to identify horses at risk, or monitor treatment success, such as diet changes. Weight tapes have been shown to overestimate weight of horses^34^, ^35^ and therefore, the animals in our study may have been marginally lighter, on average, than what is presented in Table 1. However, change in BW is what often dictates owner management, not actual weight. Therefore, especially where weighbridge scales are unavailable, use of a weight tape, as employed in our study, represents a practical monitoring tool compatible with typical field conditions.

The frequency of ID in this population of Finnhorses was 16%, which is close to previously published reports of 18% to 27% in other breeds.^12^, ^13^ Despite phenotypic markers of obesity being significantly higher in the ID group, the frequency of ID was low compared to the percentage of overconditioned or obese Finnhorses (60%). This observation supports previous findings of a lower breed representation of laminitis in this breed.^14^ Arbitrarily decreasing the cutoff to 50 μIU/mL increased the number of horses with ID to 24 (19%; 95% CI, 13%‐26%). This possibly could be a more sensitive cutoff for samples analyzed using the Immulite 2000XPi.

Fasting or resting blood glucose measurement is not a useful diagnostic test to determine ID. Instead, it should be used as part of a comprehensive diagnostic plan.^6^ Although fasting blood glucose concentration was a risk factor for ID in univariable analysis, the concentrations of all horses were within the normal reference range.

We used a higher corn syrup dose (0.45 mL/kg), which has been suggested to have higher sensitivity for ID than the previously used lower doses.^11^ However, the data originates from a study in which the purpose was to differentiate previously laminitic and nonlaminitic ponies from each other, not to find ID animals in a random population.^11^ Therefore, this higher dose may be suboptimal, and more research is warranted in this area.

## 5 CONCLUSIONS

In this sample population of Finnhorses, obesity was shown to be associated with ID. Several phenotypic indicators of obesity were found to be significantly higher in horses with ID in univariable analysis, suggesting that generalized obesity is associated with ID in cold‐blooded type horses. The frequency of ID in this population when tested using 0.45 mL/kg OST was 16%. Because owners were found to underestimate the BCS of their horses, they should be encouraged to regularly measure and record BCS and weight estimates to track changes over time.

## CONFLICT OF INTEREST DECLARATION

Authors declare no conflict of interest.

## OFF‐LABEL ANTIMICROBIAL DECLARATION

Authors declare no off‐label use of antimicrobials.

## INSTITUTIONAL ANIMAL CARE AND USE COMMITTEE (IACUC) OR OTHER APPROVAL DECLARATION

The study protocol was approved by the National Animal Experimentation Board of Finland (ESAVI/6728/04.10.07/2017).

## HUMAN ETHICS APPROVAL DECLARATION

Authors declare human ethics approval was not needed for this study.
